# Trends and focuses of hantavirus researches: a global bibliometric analysis and visualization from 1980 to 2020

**DOI:** 10.1186/s13690-022-00973-5

**Published:** 2022-10-01

**Authors:** Xiao Wei, Xinlou Li, Shuxuan Song, Xiaohui Wen, Tiezhi Jin, Chenxi Zhao, Xubin Wu, Kun Liu, Zhongjun Shao

**Affiliations:** 1 Department of Epidemiology, Ministry of Education Key Lab of Hazard Assessment and Control in Special Operational Environment, School of Public Health, Air Force Medical University, 169 Chang-Le Street, Xincheng District, Xi’an, Shaanxi 710032 People’s Republic of China; 2 Department of Medical Research, Key Laboratory of Environmental Sense Organ Stress and Health of the Ministry of Environmental Protection, PLA Strategic Support Force Medical Center, Beijing, People’s Republic of China; 3 Shaanxi Institute of Zoology, Xi’an, Shaanxi People’s Republic of China

**Keywords:** Hantavirus, Bibliometric analysis, VOSviewer, Hot topics, Research trends

## Abstract

**Background:**

There have been worldwide changes in the researches on hantaviruses in the past several decades. Nevertheless, there are few bibliometric analysis studies this field. We aim to evaluate and visualize the research focuses and trends of this field using a bibliometric analysis way to help understand the developmet and future hotspots of this field.

**Material and methods:**

Publications related to hantavirus studies were culled from the Web of Science Core Collection to generate trend analysis. The articles and reviews were re-extracted and Countries, institutions, authors, references and keywords in this field were visually analyzed by using VOSviewer and CiteSpace.

**Results:**

A total of 4408 studies were included and the number of publications regarding hantaviruses significantly increased yearly. Three thousand seven hundred sixteen research articles and reviews were retrieved to generate bibliometric analysis. These studies mainly come from 125 countries led by USA and China and 3312 institutions led by the University of Helsinki. Twelve thousand five hundred twenty nine authors were identified and Vaheri A were the most influential author. Journal of Virology was the journal with the most studies and citations. After analysis, Hemorrhagic fever with renal syndrome, Hantavirus cardiopulmonary syndrome, nephropathia epidemica and related genotypes, clinical symptoms and rodents were the most common keywords and developing areas.

**Conclusion:**

Research on hantavirus is flourishing. Cooperation among different countries and institutions in this field must be strengthened in the future. The ecology and clinical symptoms of new genotypes, the vaccine development and factors that affect host population distribution and density are current and developing areas of study.

## Background

Hantaviruses, which are members of the *Bunyaviridae* family, are enveloped, single-stranded negative-sense RNA viruses [1, 2]. hantavirus infections in humans can lead to two clinical syndromes: hemorrhagic fever with renal syndrome (HFRS) in Europe and Asia (caused by Old World hantaviruses) and Hantavirus cardiopulmonary syndrome (HCPS) in North America and South America (caused by New World hantaviruses) [3, 4]. Although China has records of a disease in its literature from 900 years ago that suggest hantavirus infections [5], the first documented pathogenic hantavirus infection in Asia was along the Hantaan River in South Korea during 1976 [6, 7]. In 1993, researchers first described HCPS in the United States, followed by identification of the Sin Nombre virus (SNV) as the etiological agent [8, 9]. During recent decades, hantavirus infections has become a globally distributed, natural-focal disease, and these viruses have gained worldwide attention as emerging zoonotic pathogens. More than 200,000 cases of hantavirus disease occur globally each year and the fatality rate is up to 12% for HFRS and 40% for HCPS, depending on the viral species [10, 11]. Hantaan virus (HTNV), Seoul virus (SEOV), Dobrava-Belgrade virus (DOV), and Puumala virus (PUUV) are typical Old World hantaviruses that are most prevalent in Europe and Asia [12]. The Sin Nombre virus (SNV) and Andes virus (ANDV) are typical New World hantaviruses that are most prevalent in North America and South America [13].

With the recent rapid increase in globalization and global warming, The hantavirus diseases have shown some new epidemic characteristics [14]. The overall number of patients infected with hantavirus has risen greatly and the prevalence is highly variable over time with the discoveries of many new genotypes, making this disease a potential threat for global public health [15]. The number of related publications has growing rapidly in recent years. Therefore, it is significant for researchers to explore and understand the most active areas of research on hantaviruses and hantavirus diseases, and to identify changes in research focus over time. However, few studies have been conducted on the trends and hot topics of hantaviruses and correlative diseases through relevant publications. Bibliometrics analysis has been widely used to explore the knowledge structure and development trends using qualitative and quantitative analysis [16, 17]. VOSviewer and CiteSpace, two novel bibliometrics software systems can not only provide researchers easier methods to quickly evaluate the distribution of countries, institutions, authors, and journals in a specific research field, but also grasp the focus and development trends in the research fields [18]. These analytical approachs has been used in diverse disciplines to develop guidelines,evaluate research hotspots, explore research trends [19].

The present study aims to explore the focuses and development trends of hantaviruses and hantavirus diseases in the past 40 years, and visualize knowledge structure with VOSviewer and CiteSpace, so as to provide a basis for future research in this field.

## Material and methods

### Data collection

**Fig. 1:**
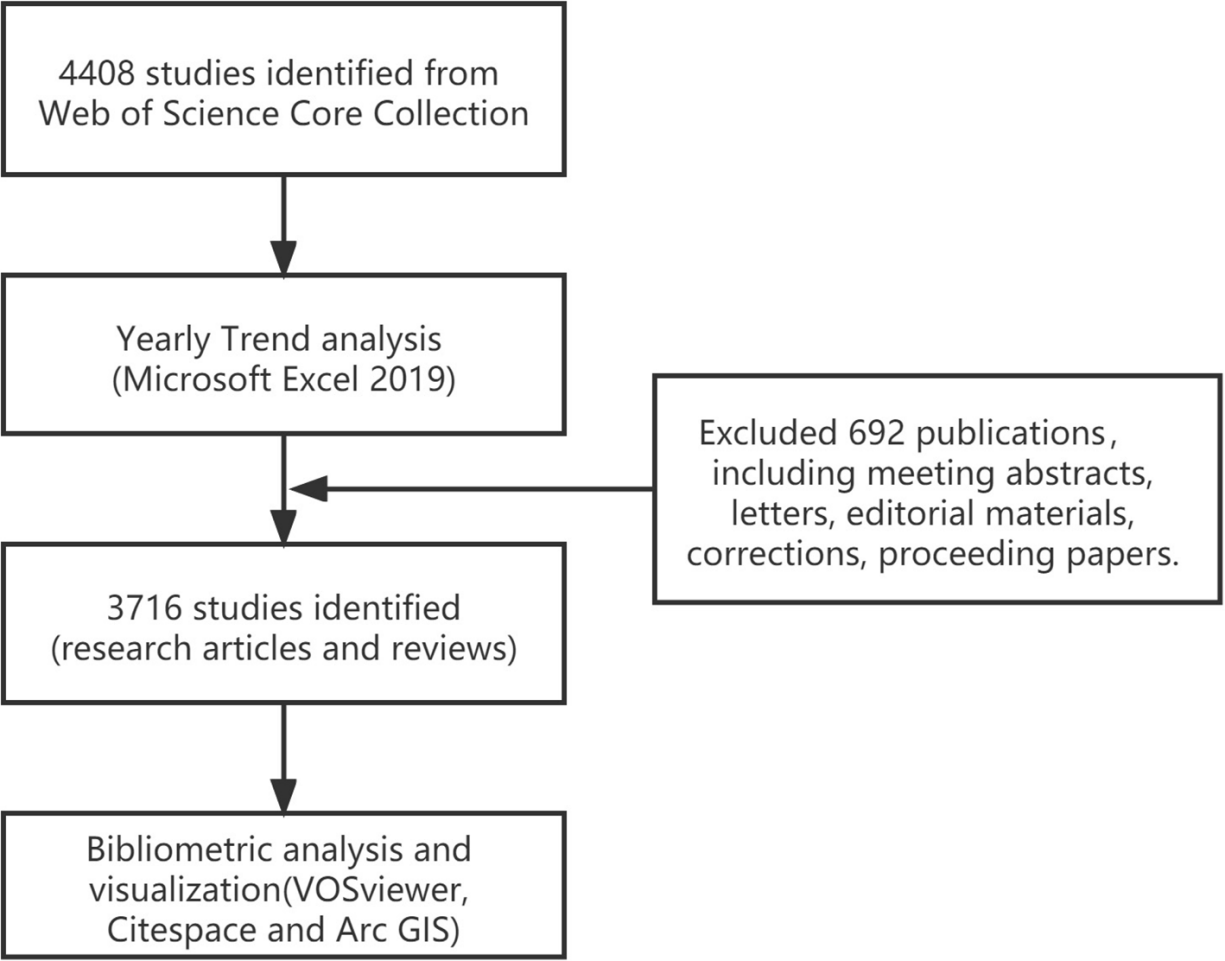
Flowchart of publications selection

Web of Science Core Collection was a database which not only contains titles, authors, institutions of the authors, countries and regions of the authors, keywords, publication years that can be used for bibliometric analysis of publications, but particularly includes the information of references that is not included in other databases. On December 31, 2020, a publication search strategy was performed through advanced search in Web of Science Core Collection: TS (topic search) = (“hamorrhagic fever with renal syndrome “ OR “hantavirus cardiopulmonary syndrome “ OR “Nephropathia epidemic” OR “Hantaan virus” OR “Seoul virus” OR “Puumala virus” OR “Sin Nombre virus” OR “Andes virus” OR “Dobrava-Belgrade virus” OR “hantavirus*”). Citation Indexes: Science Citation Index Expanded, Social Sciences Citation Index, Emerging Sources Citation Index. Timespan: 1980–01-01 to 2020–12-31. A total of 4408 studies were included in this field which prepared for trend analysis. And then 692 publications including meeting abstracts, letters, editorial materials, corrections, proceeding papers which could not provide useful information (countries of authors, institutions of authors, key words, references and the like) for bibliometric analysis were excluded. Three thousand three hundred thirty eight research articles and 368 reviews were left to produce bibliometric analysis. All data were saved in text file (Fig. 1).

### Data analysis and visualization maps

All valid data retrieved from Web of Science Core Collection were imported to Microsoft Excel 2019, VOSviewer, CiteSpace and Arc GIS to perform visual analysis.

VOSviewer is a bibliometric network analysis program developed by the Center for Science and Technology Research at Leiden University. It can be used to contruct network maps of academic publications, journals, countries, authors, and keywords based on bibliometric data. provide visual analysis and build network maps based an bibliometric data [20, 21]. These results provide a viewer for a comprehensive and detailed information of bibliometric analysis. In particular, VOSviewer is useful to display large-scale bibliometric data in an easy-to-explain way. The aim of using VOSviewer is to analyze the scienometric network and provide visualization network maps, and finally help a viewer to have a deep and comprehensive understanding the structure of the scientific research [22, 23].

CiteSpace is a bibliomertic citation visualization software developed by Professor Chen Chaomei [24, 25]. It focuses on the analysis of the potential scientific knowledge contained in the research literature and it is gradually used to visualize research focuese, evaluate the basis of scientific filed and forecast the research trends using data mining, information analysis. Knowledge maping is a novel field of information technology. CiteSpace is an effective method to analyze and visualize big bibliometric data [26].

We used Microsoft Office 2019 to analyze the trend of the number of publications to analyze the trend of the number of studies published in the year and used Arc GIS to visualize the spatial distribution of the publications [27]. And then VOSviewer and CiteSpace were used to generate bibliometric analysis, including analysis of authors, institutions, countries and regions, journals, references, key words cluster analysis and timelines.

## Results

### Overview of global trends of publication outputs

**Fig. 2:**
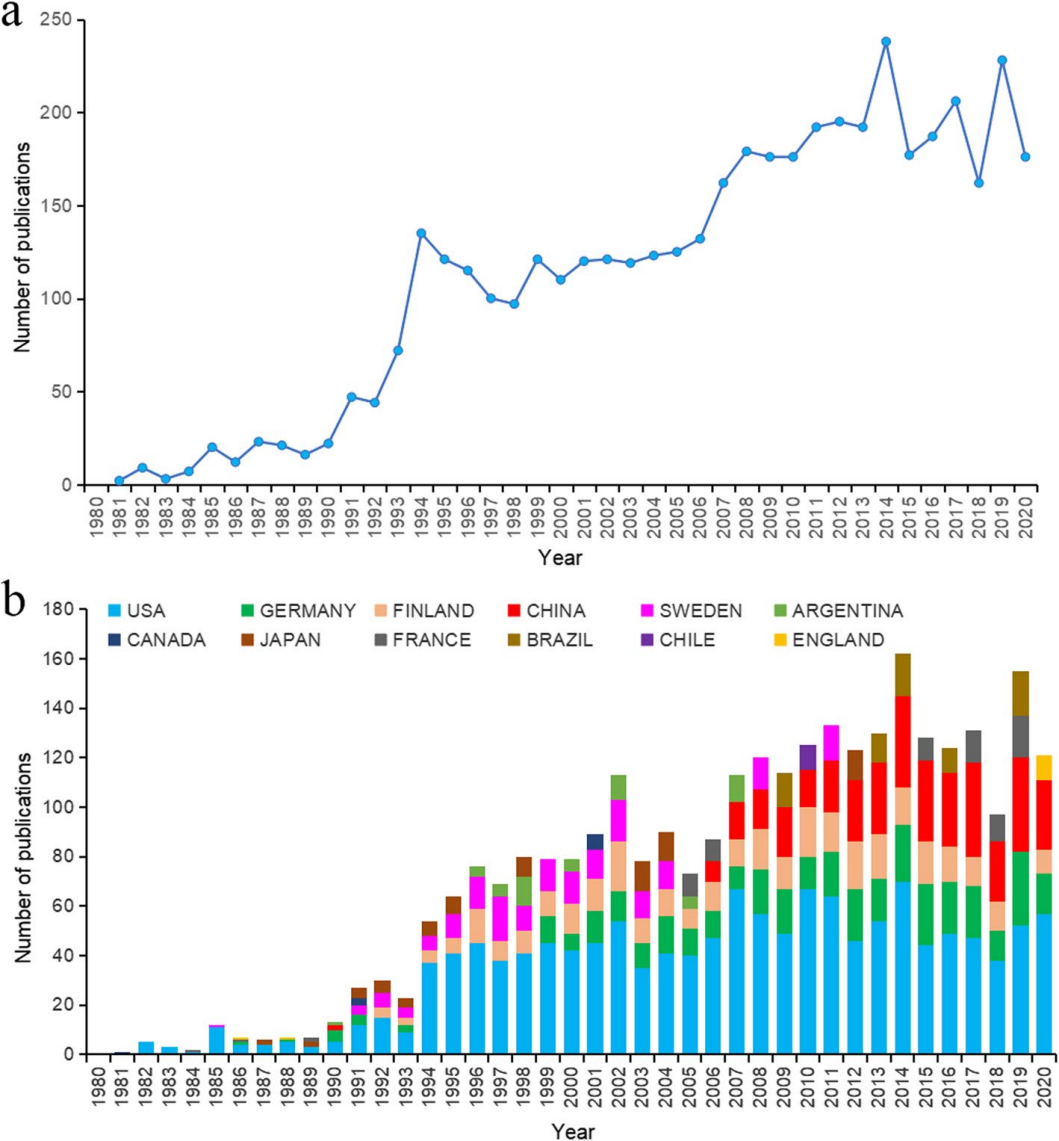
Number of publications on Hantaviruses from 1980 to 2020. **A** Annual number of all types of publications from 1980 to 2020. **B** Annual top 5 countries that had the most publications on Hantaviruses from 1980 to 2020

The number of studies published in a period reflects the trends of research in this field. Our search identified 4408 publications on hantaviruses from the WOS core database between 1980 and 2020. These publications included original research articles (3338, 75.7%), review articles (378, 8.6%), meeting abstracts (234, 5.3%), letters (204, 4.6%), and other forms of publications, such as editorial materials (108, 2.5%) and papers from proceedings (72, 1.6%). The annual number of publications increased from 2 in 1981 to 173 in 2020 (Fig. 2A). Before 1993, the annual number of publications was less than 100, and this number increased slowly over time. After 1994, the annual number of publications increased rapidly, which means that this field of hantaviruses began to receive attention of scholars. After 2000, there were more than 100 annual publications, with high variability among years since 2010. The largest number of annual publications was 234 during 2014.

### Distribution of countries/regions and institutions

**Fig. 3:**
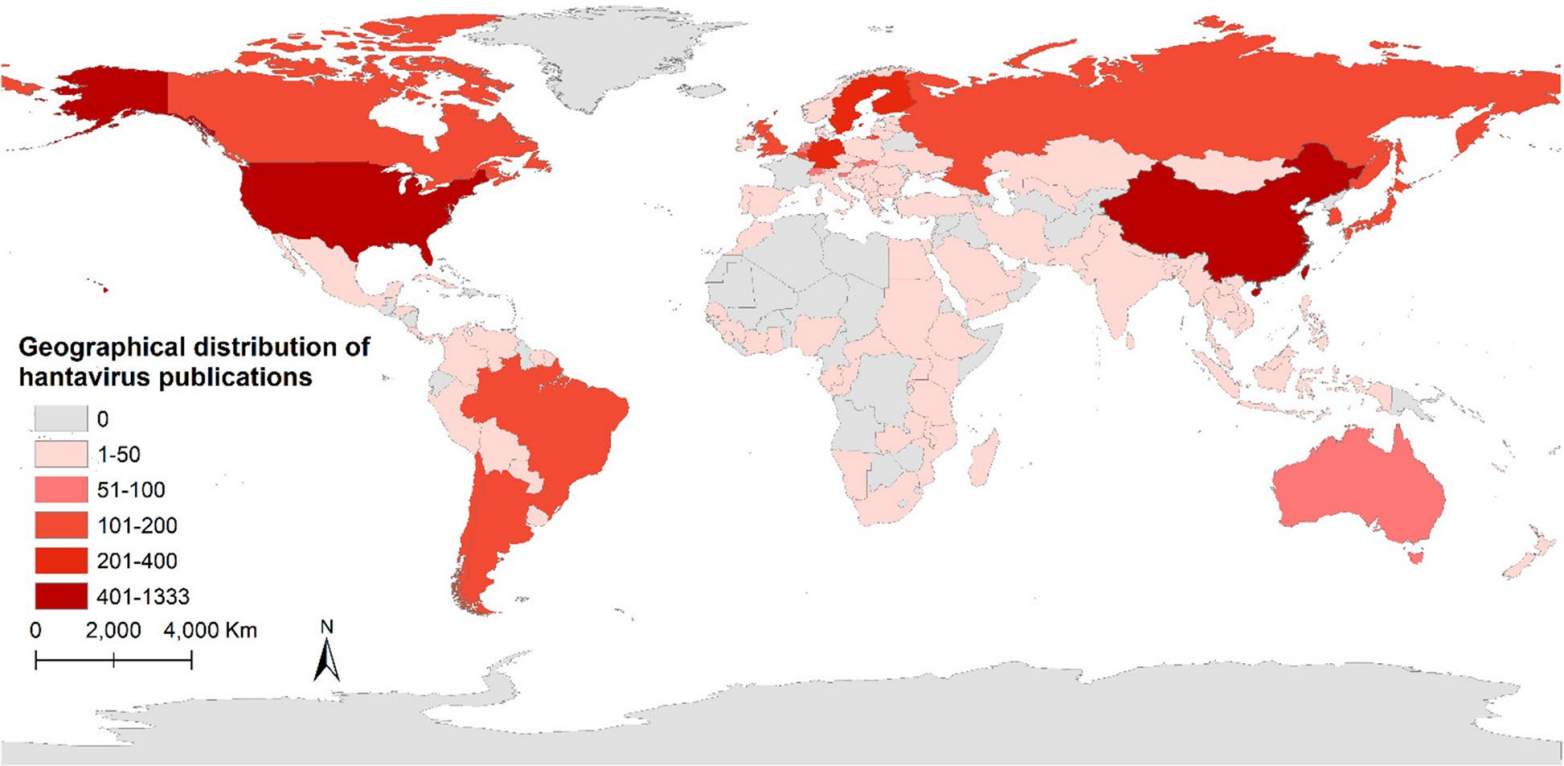
Geographical distribution of Hantavirus publications

**Fig. 4:**
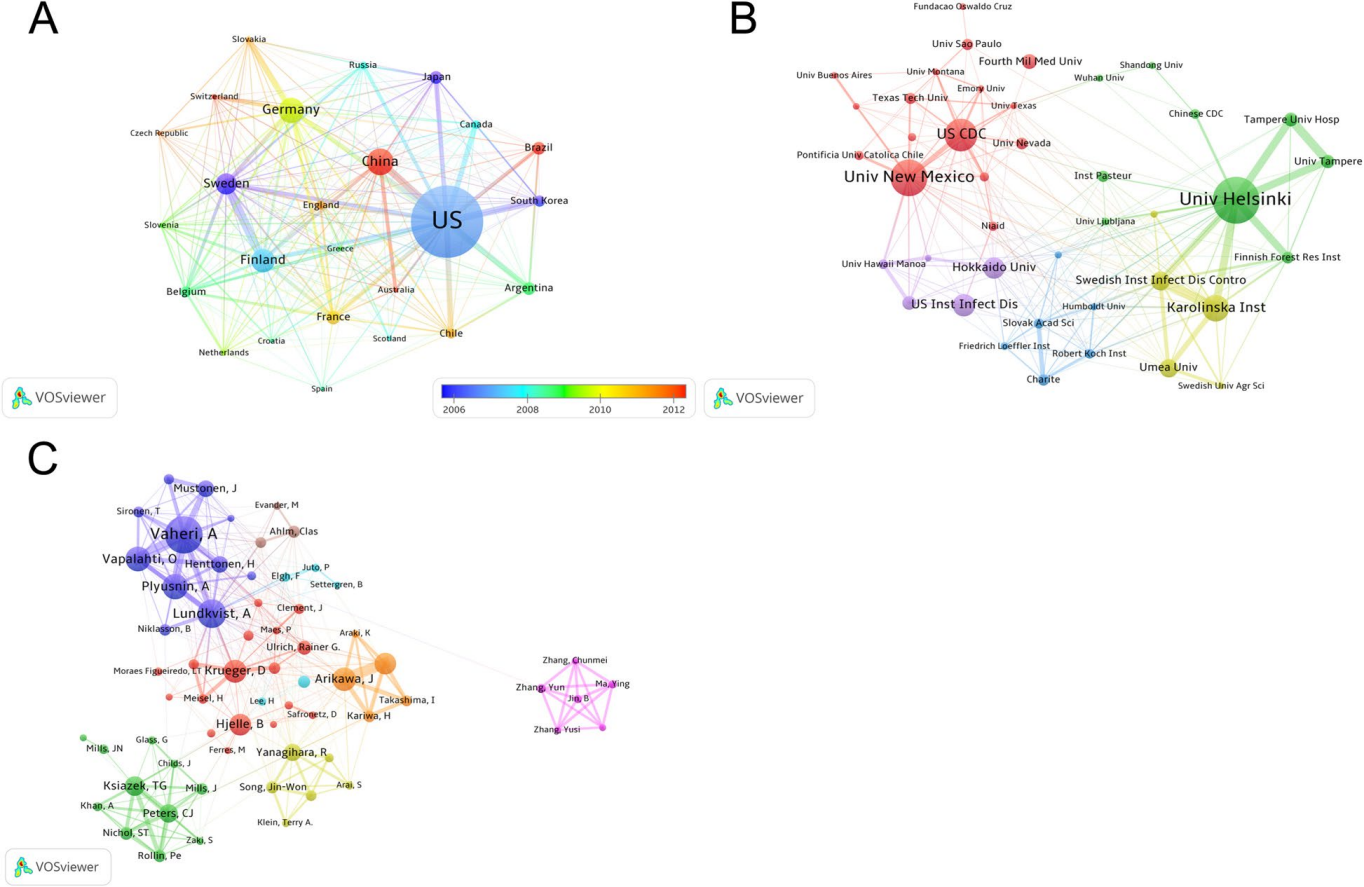
Co-authorship networks in Hantavirus research. **A** Co-authorship network map of countries that had at least 30 publications on Hantaviruses. Node color (bottom right) indicates the most active time period. **B** Co-authorship network map of institutions that had at least 30 publications on Hantaviruses. The 5 clusters are indicated by color. **C** Co-authorship network map of authors who had at least 20 publications on Hantaviruses

A total of 3716 publications (3338 research articles and 378 reviews) were indexed from 125 countries/regiions and 3312 institutions since 1980. Figure 2B indicates that the United States was the country with the most number of publications for hantaviruses from 1990 to 2020. Before 2006, top 5 countries with the publications of hantaviruses every year mainly were Japan, European and American countries. After 2006, the number of studies from China gradually increased every year. After 2011, China was second only to the United States. Figure 3 shows spatial distribution of the publications and these top countries were almost in North America, Asia, Europe, and South America. As is shown in Table 1, the most significant number of publications came from the USA (1333, 35.9%) and China (402, 10.8%), followed by Germany (379, 10.2%), Finland (343, 9.2%), Sweden (289, 7.8%), and the University of Helsinki ranked first, with 288 publications, followed by the University of New Mexico (222 publications) and the Centers for Disease Control Prevention of the United States (193 publications). Analysis of the top 10 institutions indicated 4 were in Europe, 3 were in the United States, and 3 was in Asia and this results was consistent with the spatial distribution of countries. Figure 4A, B show that the top 10 countries/institutions were the center of network maps which were visualized by the countries that had at least 30 publications and the productive institutions that published at least 30 papers. Each node represents a country or institution, the size of the node repersents the number of articles published. The line between nodes represent cooperation between countries or institutions; the more coarse line means closer cooperation. The color in Fig. 4A represents timeline and represents cluster in Fig. 4B. These maps suggest that there is active cooperation among countries and institutions, including USA, China, Germany, Finland, Sweden, the University of Helsinki, the University of New Mexico, the US Centers for Disease Control Prevention, and these countries and institutions may have played a critical role in hantaviruses research. China and Brazil are the most active countries in the past decade in this field.

**Table 1:** Countries and institutions that had the most publications on hantaviruses from 1980 to 2020

Rank	Country (Continent)	Publications (n)	Institution (Country)	Publications (n)
1	USA (North America)	1333	University of Helsinki (Finland)	289
2	China (Asia)	402	University of New Mexico (USA)	222
3	Germany (Europe)	379	US Centers for Disease Control Prevention (USA)	193
4	Finland (Europe)	343	Karolinska Institute (Sweden)	153
5	Sweden (Europe)	289	Hokkaido University (Japan)	118
6	France (Europe)	181	Medical Research Institute of Infectious Disease (USA)	116
7	Brazil (South America)	178	Swedish Institute for Infectious Disease Control (Sweden)	102
8	Argentina (South America)	169	Umea University (Sweden)	93
9	Japan (Asia)	165	Fourth Military Medical University (China)	78
10	South Korea (Asia)	145	Korea University (South Kora)	74

### Journals and co-cited academic journals

The 3716 publications were published in 821 academic journals, 10 journals published over 71 papers, and 6 of these 10 journals were based in the United States (Table 2). The Journal of Virology published the most papers (*n* = 175, IF2020 = 5.103, Q2), followed by Emerging Infectious Diseases (*n* = 138, IF2020 = 6.883, Q1).

**Table 2:** Journals that had the most publications and co-cited on hantaviruses

Rank	Journal (Country)	Count	IF2020	Q	Co-cited journal (Country)	Citations	IF2020	Q
1	Journal of Virology (USA)	175	5.103	Q2	Journal of Virology (USA)	10,100	5.103	Q2
2	Emerging Infectious Diseases (USA)	138	6.883	Q1	Emerging Infectious Diseases (USA)	7039	6.883	Q1
3	American Journal of Tropical Medicine and Hygiene (USA)	107	2.345	Q3	Virology (USA)	5841	3.616	Q3
4	Journal of General Virology (England)	104	3.891	Q3	Journal of Infectious Diseases (England)	5720	5.226	Q2
5	Virus Research (Netherlands)	100	3.303	Q3	American Journal of Tropical Medicine and Hygiene (USA)	4687	2.345	Q2
6	Journal of Medical Virology (USA)	92	2.327	Q4	Journal of General Virology (England)	4549	3.891	Q3
7	Viruses-Basel (Switzerland)	88	5.048	Q2	Lancet (England)	2956	79.321	Q1
8	Vector-Borne and Zoonotic Diseases (USA)	86	2.133	Q4	Journal of Medical Virology (USA)	2699	2.327	Q4
9	Virology (USA)	81	3.616	Q3	Proceedings of the National Academy of Science of the United States of America (USA)	2696	11.205	Q1
10	Archives of Virology (Germany)	78	2.574	Q4	Archives of Virology (Germany)	2501	2.574	Q4

*IF2020*: Impact factor in 2020, *Q*: Quartile in category

We also performed an analysis of journal co-citations and the influence of a journal depends on its co-citation ferquency, which reflects the impact of a journal in a scientific field. Among 13,138 co-cited journals, 6 journals had more than 3000 citations, and 3 of them were based in the United States. The Journal of Virology (*n* = 10,100, IF2020 = 5.103, Q2) had the most co-citations, followed by Emerging Infectious Diseases (*n* = 7039, IF2020 = 6.883, Q1). Among the top 10 co-cited journals, Lancet had the highest IF (IF2020 = 79.321).

**Fig. 5:**
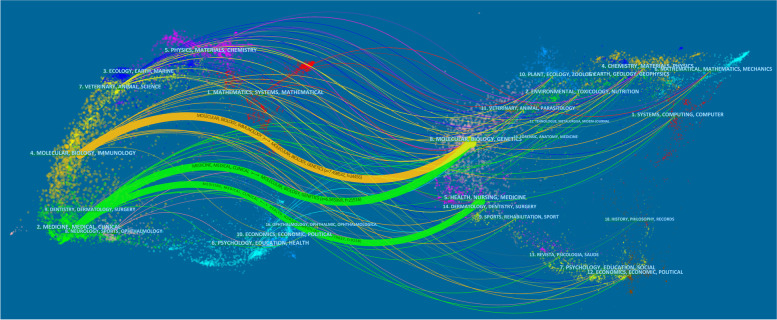
Dual-map overlay of the relationships of journals that had publications on Hantaviruses. The left part of the map was was study clusters of journals which references were published in, the right research classifications of journals which original article is published in, the colorful paths represented cited relationships

A dual-map overlay indicated that the main active research areas of 3716 publications and relevant research were molecular biology, immunology, clinical medicine, genetics and health nursing medicine. There were three main paths (indicated by the 1 orange path and 2 green paths in Fig. 5). The orange path represented studies published in the journals of molecular biology and genetics that were usually cited by publications in the journals of molecular biology and immunology. The green paths represented studies that were published in the journals of molecular biology and genetics and the journals of health nursing medicine that were usually cited by publications in the journals of clinical medicine.

### Authors and co-cited authors

There were 12,529 authors of thes publications on hantaviruses. As shown in Table 3, Vaheri. A had the highest number of publishend literatures (209), followed by Lundkvist. A (146) and Plyusnin. A(125). Co-cited authors means two or more authors who were cited by another paper at the same time,these two or more authors have co-cited relationship. Among 41,526 co-cited authors, Schmaljohn. C (2354) was the most frequently cited author, followed by Lee. HW(1726) and Plyusnin. A (1443). Co-authorship of all authors who had at least 20 publications was visualized and Fig. 4C shows that these top authors were at the center of the network map. Each node represents an author,with larger cicle meaning more articles. Thicker lines means closer cooperation between two authors. Different colors represent different cluster of the cooperation. These results probably means that these top authors were the influencial scholars in this field and had closer cooperations with other authors.

**Table 3:** Top 10 Authors and co-cited authors on hantaviruses

Rank	Author	Publications (n)	Co-cited Author	Co-citations (n)
1	Vaheri A et al	209	Schmaljohn C et al	2354
2	Lundkvist A et al	146	Lee HW et al	1726
3	Plyusnin A et al	125	Plyusnin A et al	1443
4	Vapalahti O et al	120	Hjelle B et al	1064
5	Arikawa J et al	108	Mills JN et al	1040
6	Krueger D et al	105	Lundkvist A et al	1006
7	Hjelle B et al	98	Vapalahti O et al	972
8	Yoshimatsu K et al	98	Nichol ST et al	841
9	Ksiazek TG et al	84	Klempa B et al	786
10	Peters CJ et al	78	Childs JE et al	743

### Basic knowledge and hot topics in hantavirus research

**Fig. 6:**
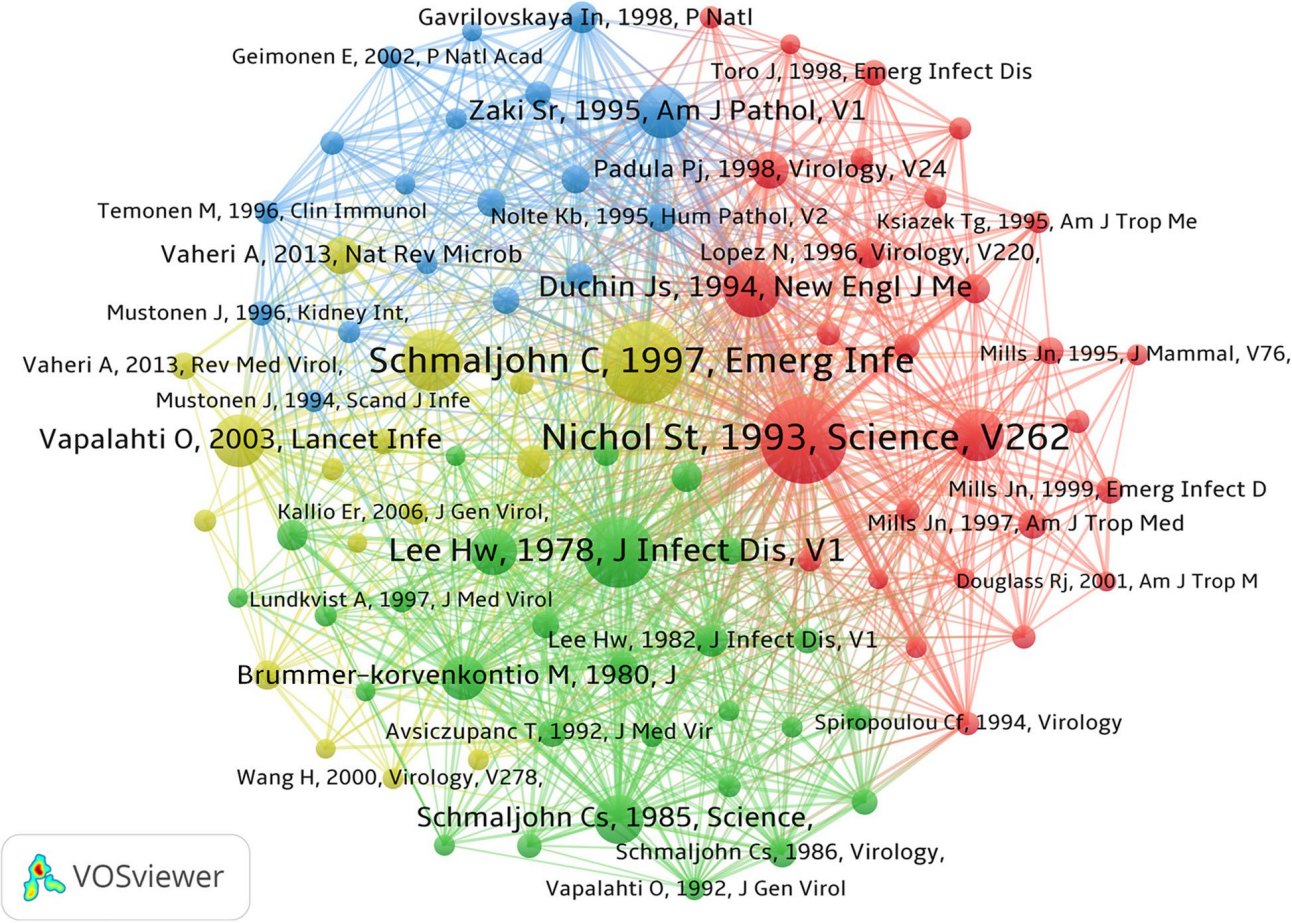
Network map of co-citation references of publications on Hantaviruses that were cited at least 100 times

References represent the basic knowledge of a a specific research field. Analysis of the 3716 publications indicated there were 64,713 references, with an average of 17 references per publication. On December 31, 2020, we compiled a list of the 10 most frequently cited references related to research on hantaviruses (Table 4). Among these cited references, 92 references were cited more than 100 times, and the references listed in the top 10 were all cited more than 300 times. The most cited paper was Genetic identification of a hantavirus associated with an outbreak of acute respiratory illnes, a genetic analysis of a new serotype of hantaviruses reported in the southwestern United States. We also analyzed the top 90 references (each of which was cited at least 100 times) to generate a network map (Fig. 6). This network showed 4 clusters (indicated by 4 colors). suggesting that these main references represents four main fields of research.

**Table 4:** Most-cited publications on hantaviruses

Rank	First author (year)	Journal	Title	Citations (n)
1	Nichol ST et al. (1993) [28]	Science	Genetic identification of a hantavirus associated with an outbreak of acute respiratory illness	653
2	Schmaljohn C et al. (1997) [2]	Emerging Infectious Diseases	Hantaviruses: A global disease problem	622
3	Lee HW et al. (1978) [7]	The Journal of Infectious Diseases	Isolation of the etiologic agent of Korean hemorrhagic fever	492
4	Jonsson CB et al. (2010) [12]	Clinical Microbiology Reviews	A global perspective on Hantavirus ecology, epidemiology, and disease	450
5	Duchin JS et al. (1994) [9]	New England Journal of Medicine	Hantavirus pulmonary syndrome: A clinical description of 17 patients with a newly recognized disease	391
6	Vapalahti O et al. (2003) [10]	Lancet Infectious Diseases	Hantavirus infections in Europe	362
7	Zaki SR et al. (1995) [29]	American Journal of Pathology	Hantavirus pulmonary syndrome. Pathogenesis of an emerging infectious disease	355
8	Childs JE et al. (1994) [30]	The Journal of Infectious Diseases	Serologic and genetic identification of Peromyscus maniculatus as the primary rodent reservoir for a new Hantavirus in the southwestern United States	349
9	Schmaljohn CS et al. (1985) [1]	Science	Antigenic and genetic properties of viruses linked to hemorrhagic fever with renal syndrome	305
10	Plyusnin A et al. (1996) [31]	The Journal of General Virology	Hantaviruses: Genome structure, expression and evolution	300

**Fig. 7:**
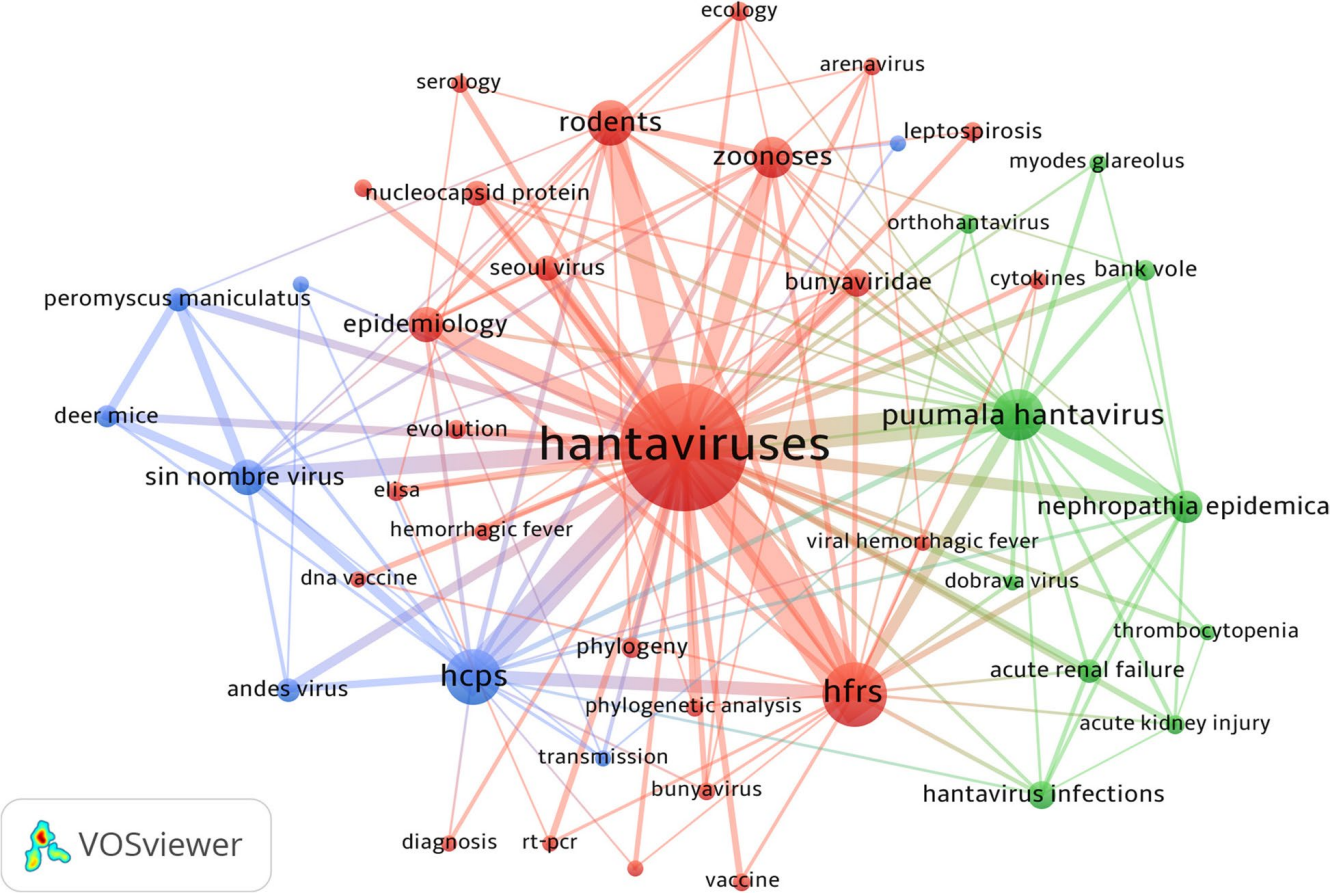
Network map of keywords in publications on Hantaviruses that occurred at least 20 times

**Fig. 8:**
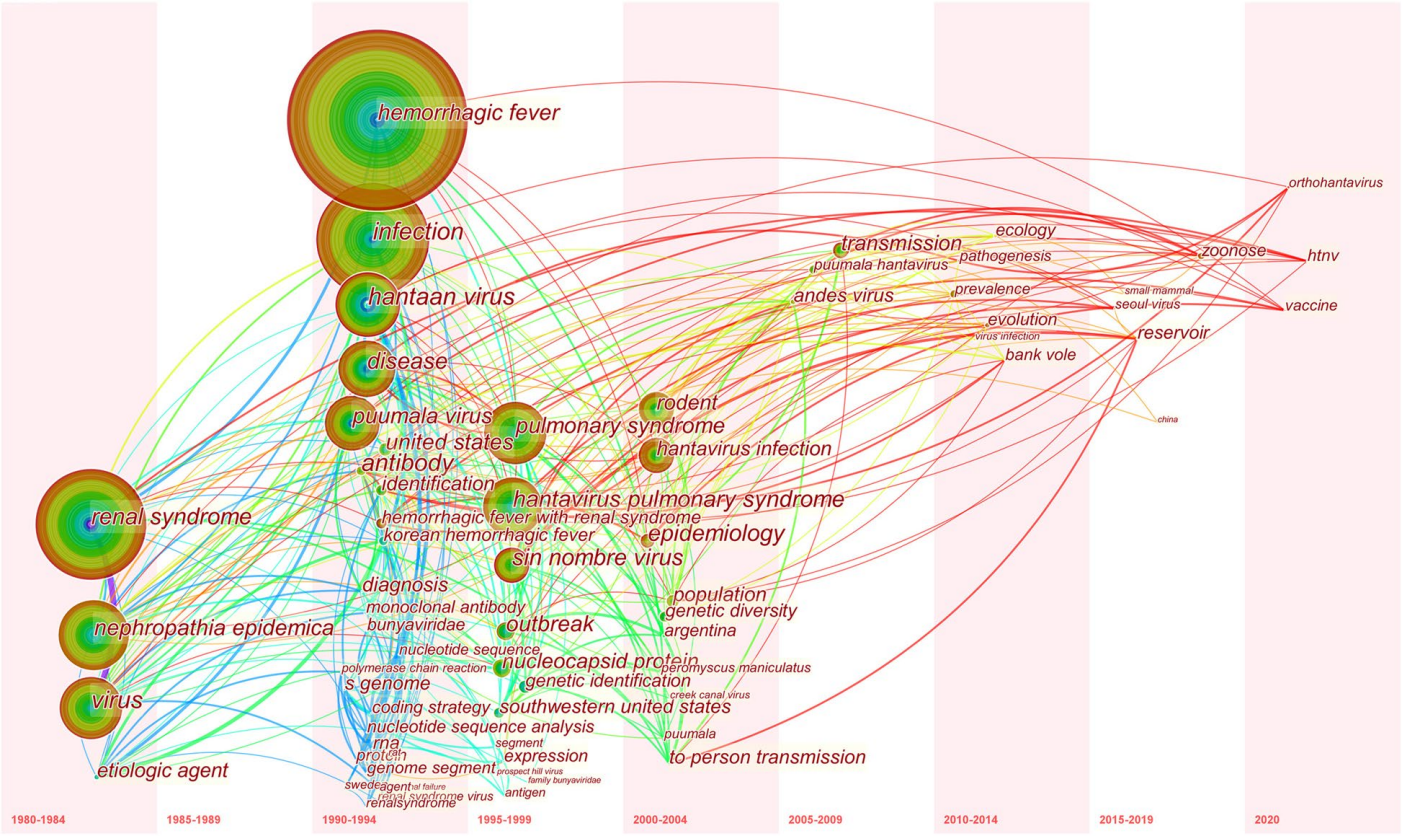
Time-zone map of the top 20 keywords in publications on Hantaviruses during 4-year intervals from 1980 to 2020

Keywords are the core of a published article and represent the research topics. Through analyzing these keywords, we can summarize the focuses and research directions in a specific field. We used VOSviewer to cluster the keywords that occurred at least 20 times. Each node represents a keyword and the larger node represents the more repetition for a keyword. The color of node identifies the cluster to which it belongs. Figure 7 shows that these keywords were divided into 3 clusters of green, red, and blue, suggesting three research fields. Green clusters are consist of nephropathia epidemica, puumala hantavirus, bank vole, dobrava virus, thrombocytopenia. Red clusters are composed of HFRS, rodents, epidemiology, seoul virus, vaccine. The keywords of blue clusters are HCPS, andes virus, sin nombre virus, deer mice. We used Citespace to visualize a time-zone view of keywords. This kind of networkmap is designed based on the interactions between keywords, and it helps viewer to explore the evolution track and stage characteristics. Figure 8 shows that the research mainly based on the clinical syndrome and serotypes of hantaviruses from 1980 to 2000, and the main keywords are renal syndrome, hemorrhagic fever, pulmonary syndrome, hantaan virus, puumala virus. From 2000 to 2020, the research mainly focuses on the epidemiology and influence factors in this field, and the main keywords were transmission, rodents, ecology, evolution, vaccine.

## Discussion

Our study has produced an up-to-date and in-depth exploration of the focuses and development trends of hantaviruses and hantavirus diseases in the past 40 years through VOSviewer and CiteSpace, and visualize knowledge structure about the relationships of related countries, institutions, authors, keywords and references with many bibliometric analysis methods in two novel bibliometric analysis software systems, providing a basis for future research in this field.

### General information

The trend analysis indicated three major stages of research on hantaviruses: the first stage was from 1980 to 1994, the second stage was from 1995 to 2014, the third stage was from 2015 to 2019. The number of publications increased slowly from 1980 to 1992, and rapidly grew from 1992 (44 publications) to 1994 (128 publications). The second stage had variable numbers of publications over time, but the number of publications increased significantly from 2012 to 2014. The rapid growth in the number of publications during the first and second stages might due to the two major outbreaks of hantavirus diseases: HCPS in the “Four Corners” area of the United States during 1993 and HFRS in Germany during 2012 [11, 32, 33]. These publications appeared 1 to 2 years after the outbreaks. Research on hantaviruses has always been a hot spot in the United States and European countries. However, research on this topic is becoming increasingly more common in China, indicating that Chinese researchers are paying more attention to this topic.

According to the distribution of countries/regions, These results indicated the United States was the most productive and cooperative country, probably because HCPS was classified as a notifiable disease in the United States in 1995, and hantaviruses-related studies have received significantly more attention over time [29–31, 34]. Among all analyzed publications, 2927 publications were published in the United States, China, Germany, Finland, Sweden, and France. The United States, China, Germany, Finland, Sweden, France, Argentina, Japan, and Brazil were at the center of an international co-authorship network, suggesting that these countries are the current leaders in research on hantaviruses (Figs. 3 and 4A). Also, these countries are the main endemic areas of hantaviruses. In recent decades, most reported infections with hantaviruses are in Asia, but the number of cases in the United States and Europe has increased [10]. China and Brazil are the most active countries, suggesting that related cases has increased considerably over the past decade in these countries, becoming a severe public health problem. Figure 4A also shows that cooperation of countries/regions that have greater geographic proximity and cultural similarity is close, such as Sweden has more close cooperation with Finland than China. Therefore, it is strongly suggested that different geographies with different cultures should strengthen cooperation.

We found that 4 of the top 10 most productive institutions were in Europe, and 3 was in Asia. These 7 institutions focused on the HFRS, or nephropathia epidemica (NE). The other 3 top institutions were in the United States, and mainly studied HCPS. The top 10 institutions were near the core of each cluster in a map of co-authorship, indicating that these institutions lead the research trends in this field (Fig. 4B). Among the top 10 authors, 5 were from Europe, 3 were from the United States, and 2 were from Asia (Tables 3). Among all 12,529 authors, 727 published more than 5 papers in this field. The cooperation between two authors within the same co-author cluster was common, but there was much less cooperation between authors in different co-author clusters (Fig. 4C). This indicates that although many authors and institutions were interested in research on hantaviruses, collaborations among disparate authors and institutions was limited. Promoting more collaboration between disparate authors and institutions may improve the quality and increase the number of studies on hantaviruses.

We found that 6 of the top 10 journals and co-cited journals were from the United States, indicating that these journals made the most contributions and attracted the most attention. In addition, high-IF journals had more co-citations (Table 2), suggesting that these journals had the most important roles in studies of hantaviruses. The dual-map overlay of journals (Fig. 5) indicated that the relationships between journals and co-cited journals were responsible for the main research directions, thus providing indispensable information for researchers new to this field.

Seven of the top 10 most frequently cited references were published in 1994 or later. This is consistent with our findings that the number of publications increased rapidly during this time (Fig. 2A) and also indicates that the worldwide understanding and recognition of hantavirus infections has greatly increased since 1994. Bibliometric analysis of references and co-citations (Fig. 6) showed that there were 4 clusters representing basic areas of research: structure and function of hantaviruses; identification of hantaviruses; clinical symptoms and pathophysiologic studies of hantavirus diseases; and ecology and epidemiology of hantaviruses.

### The focuses and frontiers

Keywords summarize research focuses and core content of publications. Based on keyword co-occurence analysis, it possibly provides a way to learn about the distribution and development of different research focuses in a specific field. Keywords cluster analysis was conducted on the basis of co-occurence, and finally three clusters were formed.

According to cluster analysis (Fig. 7)and the time-zone analysis (Fig. 8), we determine main research focuses and frontiers in this field. The main contents are as follows:

#### HFRS and related research

Hemorrhagic fever with renal syndrome (HFRS), characterized by renal failure and haemorrhagic manifestations, is mainly caused by HTNV, SEOV, and several recently discovered species of hantaviruses [35]. The clinical symptoms of HFRS varies from subclinical, mild, and moderate to severe, depending partly on the causative serotype of the hantaviruses. In general, the clinical characteristiccs caused by HTNV or DOBV are more severe, whereas SEOV causes moderate disease and PUUV cause mild forms of disease [10, 36]. According to the latest report, there are about 15,000 to 20,000 HFRS cases worldwide annually, and the fatality rate ranges from 1 to 12% [10, 11]. Most infections are in the Asia–Pacific region and Europe, and this disease has become a serious threat for public health, especially in China [37]. A recent study reported almost 20,000 cases per year in China [38], and this accounts for more than 90% of the reported cases worldwide [39]. Apodemus agrarius and Rattus norvegicus are the main reservoir hosts for HFRS in wild and residential areas, respectively [40]. Rapid economic growth, urbanization, and climate change may have increased the transmission of this zoonotic disease by increasing rodent populations, and these factors may be the important influence effects in this field. Great changes have occurred in areas where there are epidemics, new epidemic areas have emerged, and endemic areas have gradually increased. To our knowledge, only a few inactivated vaccines are currently used in China and South Korea, and there is no universally licensed vaccine for HFRS [41]. In addition, there are no specific treatments for this disease, and most clinicians recommend supportive care.

#### HCPS and related research

Hantavirus cardiopulmonary syndrome (HCPS), characterized by pneumonia and cardiovascular dysfunction, was first reported in the United States [28]. Initial research reported that HCPS was characterized by severe, acute cardiopulmonary failure, and reported more than 1,000 cases. Compared with HFRS, HCPS is a more severe disease with a fatality rate from 35 to 50% [42, 43]. The clinical presentations of HCPS generally are three phases: prodromal, cardiopulmonary and convalescent, and clinical syndromes can vary from mild hypoxaemia to respiratory failure with cardiogenic shock [44]. SNV and ANDV are the two major causative agents, and approximately 43 strains of these viruses were reported in the Americas, 20 of which can cause HCPS. SNV is the most prevalent hantavirus genotype in North America (United States and Canada), and deer mice (Peromyscus maniculatus) are the predominant rodent reservoir. ANDV is the major cause in South America (Chile and Brazil), and this hantavirus is unique because person-to-person transmission is possible, and this has posed a major challenge to the health care systems of Argentina and Chile [45–47]. Recent research in Germany indicated that PUUV caused HCPS [48]. At present, there are also no specific treatment measures for HCPS.

#### NE and related research

Nephropathia epidemica (NE) is a mild form of HFRS, which mainly occurs in Europe, and is characterized by acute renal failure and thrombocytopenia [49, 50]. The occurence of thrombocytopenia in this type of disease varies from 39 to 98%, whereas bleeding complications are rare in acute NE [46]. It is also reported that smokers acquire more severe kidney than non-smokers [51]. NE was discovered in Sweden during the 1930s [52], but the pathogenic virus (PUUV) was first identified in bank voles (Clethrionomys glareolus) in Finland during 1980 [50]. A 2014 study reported that thousands of human hantavirus infections occurred annually, and the number of NE cases has increased during recent years due to climate change and economic development in Europe [32]. Although several hantavirus species are circulating in Europe, such as Dobrava virus, Saremaa virus, and Tula virus, PUUV is by far the most prevalent species [53]. As with other diseases caused by hantaviruses, there are no specific treatments diseases caused by these species, and only supportive care is used.

#### The changes of research frontiers

Our time-zone map of the top 20 keywords of each slice (Fig. 8) indicated that most of the initial research on hantaviruses focused on clinical symptoms and the cause of this type of disease before 1990. After then, research on identifications of serotypes of hantaavireses, such as hantaan virus, puumala virus., and pathogenesis of these diseases probably have become research frontiers in this field from 1990 to 2000. And there was a great increase in this research during the 1990s, in line with our conclusions above. From 2000 to 2010, studies on the correlations between the characteristics of diseases and the evolution of the pathogens, the transmission of these diseases have received more attention. Research on the factors affecting the prevalence of the diseases, vaccine and factors affecting the distributions of host animals have been research frontiers in this field in the past decade. Our results suggest that future studies will focus on the characteristics and distributions of hantavirus diseases, the discovery of new species, the development of vaccines, and factors that affect the distribution and density of rodent host populations.

## Limitations

There are some limitations to the study. First, in order to uniform the quality of publications and ensure the unified standards of collecting data, the data for bibliometric analysis were only extracted from WOS Core Collection, not including studies from other database such as Scopus, PubMed, MEDLINE or Google Scholar which did not include the detailed data of references. Thus, some publications appearing only through one of these other databases may have been missed [54, 55]. Second, our study may have language bias, though we did not set any restrictions on language of publications, the great mass of publications in WOS Core Collection are in English. Third, some author or institution may be missed, because their names may include special characters which may be difficult for applications to identify. Fourth, when we conducted analysis of countries for publications, the current methods have ignore the repeated records in a article, which maybe bring bias. We plan to address these by exploring new bibliometric ways in future work.

## Conclusion

We used VOSviewer and CiteSpace for a bibliometric visualization analysis of publications on hantaviruses. To our knowledge, this is the first research to use these tools to analyze hantavirus publications. We found that the USA, China, Germany, Finland, Sweden, France, Argentina, Japan, and Brazil are the countries which have attach importance to the research of of hantaviruses in this field. Institutions and authors of these countries are more influential in this research area. Different countries and institutions shoul strengthen cooperation with each other. Studies of viral function and identification, coupled with clinical symptoms and pathophysiologic studies, were the major research trends during the 1990s and early 2000s. The ecology of new genotypes and the epidemiology of hantaviruses were active areas of research during the past decade. We believe that vaccine development and factors that affect host population distribution and density will probably be hot topics in the future.

## Data Availability

All data of literatures used for analysis are available upon a proper request from the corresponding author Kun Liu at liukun5959@qq.com or Zhongjun Shao at 13,759,981,783@163.com.

## Abbreviations

USA: The United States
HFRS: Hemorrhagic fever with renal syndrome
HCPS: Hantavirus cardiopulmonary syndrome
NE: Nephropathia epidemica
HTNV: Hantaan virus
SEOV: Seoul virus
DOV: Dobrava-Belgrade virus
PUUV: Puumala virus
SNV: Sin Nombre virus
ANDV: Andes virus
