# Correction: Trends and focuses of hantavirus researches: a global bibliometric analysis and visualization from 1980 to 2020

**DOI:** 10.1186/s13690-022-00989-x

**Published:** 2022-11-17

**Authors:** Xiao Wei, Xinlou Li, Shuxuan Song, Xiaohui Wen, Tiezhi Jin, Chenxi Zhao, Xubin Wu, Kun Liu, Zhongjun Shao

**Affiliations:** 1 Department of Epidemiology, Ministry of Education Key Lab of Hazard Assessment and Control in Special Operational Environment, School of Public Health, Air Force Medical University, 169 Chang-Le Street, Xincheng District, 710032 Xi’an, Shaanxi People’s Republic of China; 2 Department of Medical Research, Key Laboratory of Environmental Sense Organ Stress and Health of the Ministry of Environmental Protection, PLA Strategic Support Force Medical Center, Beijing, People’s Republic of China; 3 Shaanxi Institute of Zoology, Xi’an, Shaanxi People’s Republic of China

**Correction to: Archives of Public Health (2022) 80:218**

10.1186/s13690-022-00973-5

Following publication of the original article [1], the authors reported some errors in the distribution of national and regional levels, and the national boundaries in Fig. 3. The correct Fig. 3 has been provided in this Correction.

**Fig. 3:**
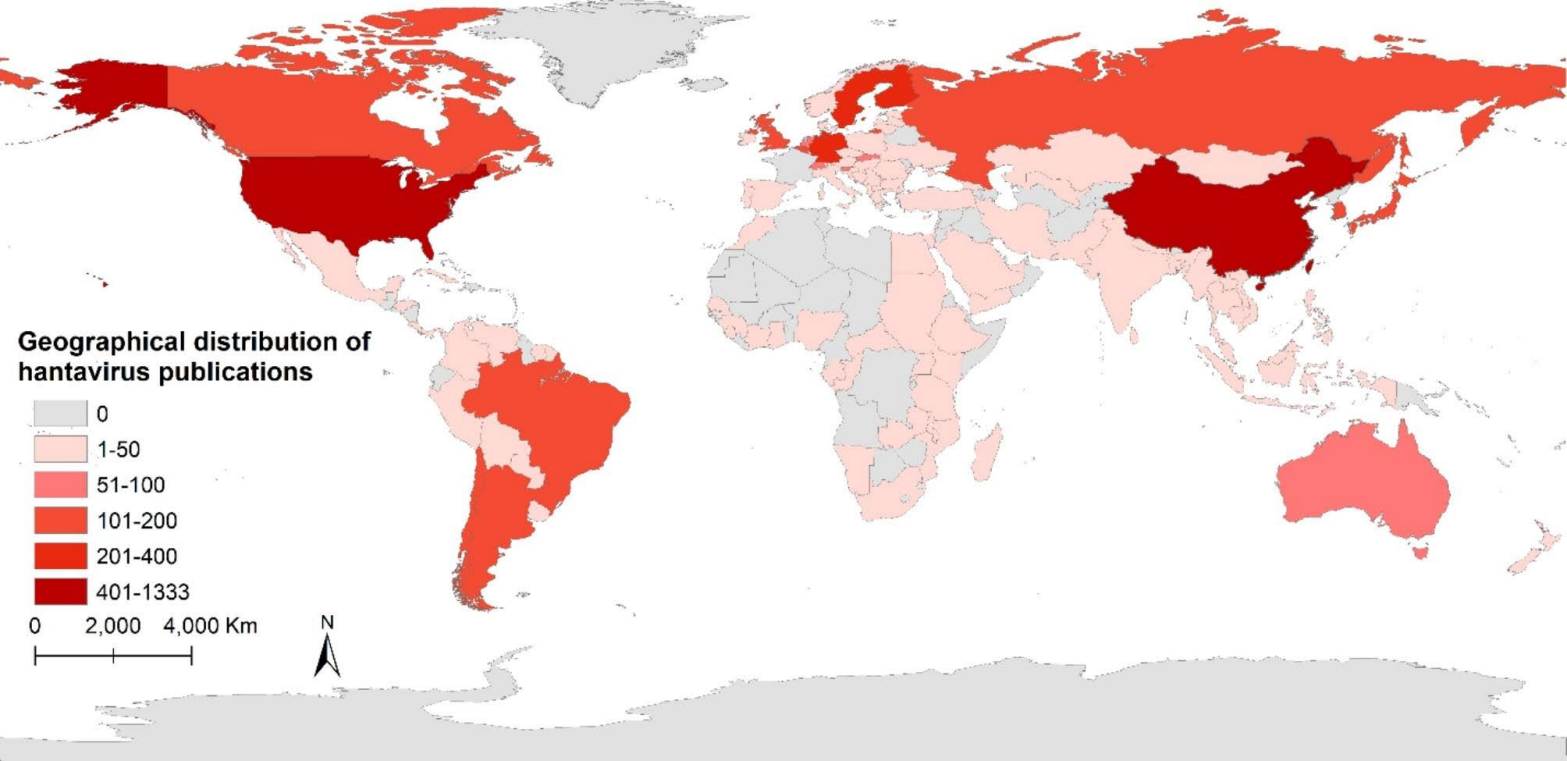
Geographical distribution of Hantavirus publications

The original article [1] has been corrected.
